# Assessing Cerebral White Matter Microstructure in Children With Congenital Sensorineural Hearing Loss: A Tract-Based Spatial Statistics Study

**DOI:** 10.3389/fnins.2019.00597

**Published:** 2019-06-21

**Authors:** Muliang Jiang, Zuguang Wen, Liling Long, Chi Wah Wong, Ningrong Ye, Chishing Zee, Bihong T. Chen

**Affiliations:** ^1^Department of Radiology, First Affiliated Hospital of Guangxi Medical University, Nanning, China; ^2^Department of Diagnostic Radiology, City of Hope National Medical Center, Duarte, CA, United States; ^3^Center for Informatics, City of Hope National Medical Center, Duarte, CA, United States; ^4^Department of Radiology, Keck School of Medicine, University of Southern California, Los Angeles, CA, United States

**Keywords:** congenital sensorineural hearing loss, diffusion tensor imaging, white matter, tract-based spatial statistics, diffusivity, magnetic resonance imaging

## Abstract

**Objectives:**

To assess the microstructural properties of cerebral white matter in children with congenital sensorineural hearing loss (CSNHL).

**Methods:**

Children (>4 years of age) with profound CSNHL and healthy controls with normal hearing (the control group) were enrolled and underwent brain magnetic resonance imaging (MRI) scans with diffusion tensor imaging (DTI). DTI parameters including fractional anisotropy, mean diffusivity, axial diffusivity, and radial diffusivity were obtained from a whole-brain tract-based spatial statistics analysis and were compared between the two groups. In addition, a region of interest (ROI) approach focusing on auditory cortex, i.e., Heschl’s gyrus, using visual cortex, i.e., forceps major as an internal control, was performed. Correlations between mean DTI values and age were obtained with the ROI method.

**Results:**

The study cohort consisted of 23 children with CSHNL (11 boys and 12 girls; mean age ± SD: 7.21 ± 2.67 years; range: 4.1–13.5 years) and 18 children in the control group (11 boys and 7 girls; mean age ± SD: 10.86 ± 3.56 years; range: 4.5–15.3 years). We found the axial diffusivity values being significantly greater in the left anterior thalamic radiation, right corticospinal tract, and corpus callosum in the CSHNL group than in the control group (*p* < 0.05). Significantly higher radial diffusivity values in the white matter tracts were noted in the CSHNL group as compared to the control group (*p* < 0.05). The fractional anisotropy values in the Heschl’s gyrus in the CSNHL group were lower compared to the control group (*p* = 0.0015). There was significant negative correlation between the mean fractional anisotropy values in Heschl’s gyrus and age in the CSNHL group < 7 years of age (*r* = −0.59, *p* = 0.004).

**Conclusion:**

Our study showed higher axial and radial diffusivities in the children affected by CNHNL as compared to the hearing children. We also found lower fractional anisotropy values in the Heschl’s gyrus in the CSNHL group. Furthermore, we identified negative correlation between the fractional anisotropy values and age up to 7 years in the children born deaf. Our study findings suggest that myelination and axonal structure may be affected due to acoustic deprivation. This information may help to monitor hearing rehabilitation in the deaf children.

## Introduction

Congenital sensorineural hearing loss (CSNHL) is a type of deafness that occurs prior to language development. Permanent childhood hearing loss due to CSNHL is estimated to be 1.2 to 1.7 cases per 1,000 live births, and up to 30% of these affected children have profound hearing loss (Kral and O’donoghue, 2010; Paludetti et al., 2012). Congenital deprivation of sound stimuli in children with CSNHL may delay language acquisition and alter the development of auditory neural pathways, resulting in brain structural changes (Chari and Chan, 2017). Delayed diagnosis of hearing loss in infants and young children with CSNHL results in severe deficits in learning and development. In addition, severe-to-profound hearing loss causes a severe societal burden due to reduced work productivity and the need for expensive special education resources for children with prelingual deafness (Mohr et al., 2000). Cochlear implant being the most effective treatment for CSNHL should be placed within the first 3.5 to 4 years of life when the developing brain has the maximal plasticity for reorganization in order to optimize cognitive functioning (Kral and Sharma, 2012). Nevertheless, cochlear implant placed late or even in adult age may enable the patients to hear and could help to compensate for the deficits in the brain development. However, prior studies have shown that individuals with the cochlear implant placed late or in adult age may have persistent auditory deficits, and they may not gain effective speech understanding even with the cochlear implant for a long period of time (Kral and Sharma, 2012). Therefore, it is prudent to diagnose and treat children with CSNHL early in life to improve their learning and development outcomes.

Neuroimaging studies have contributed to our understanding of brain alterations and neuroplasticity in deafness. Prior studies of macrostructural differences in deaf people, compared to control participants without hearing loss, showed lower white matter (WM) volume but preserved gray matter volume in the auditory region, including the Heschl’s gyrus (HG) and the adjacent temporal lobe (Hribar et al., 2014). In addition to the reported WM macrostructural changes, there are also WM microstructural alterations in people with hearing loss (Kim et al., 2009; Miao et al., 2013; Park et al., 2018). However, less is known about the WM microstructural properties specifically in children with CSNHL.

Diffusion tensor imaging (DTI) has been commonly used to study WM microstructure. DTI is an established magnetic resonance imaging (MRI) method that measures the directionality of water diffusion in the brain and is more sensitive for detecting WM microstructural alterations than volumetric methods (Douaud et al., 2011). A prior DTI study found abnormal WM microstructure in adolescents with prelingual deafness, compared to healthy control adolescents (Park et al., 2018). Their study found lower fractional anisotropy (FA) values in the WM tract of the HG and inferior fronto-occipital fasciculus (IFOF), among other WM areas, in deaf children younger than 4 years but not in older children. However, another DTI study showed lower FA values and higher radial diffusivity (RD) in bilateral superior temporal gyri, HG, and splenium of the corpus callosum in older children (10–18 years of age) with prelingual deafness (Miao et al., 2013). These results indicate that WM microstructural properties may be different in children with hearing loss, but there is limited information about how brain alters in children affected by CSNHL. Furthermore, the existing study results are inconsistent, leaving a gap in knowledge.

To address the knowledge gap, we designed the current study to test the hypothesis that children with CSHNL have different WM microstructural properties compared to the healthy controls with normal hearing. We evaluated the cerebral WM microstructural properties using two different methods, including a whole-brain voxel-wise tract-based spatial statistics (TBSS) method and a region of interest (ROI) approach focusing on the auditory cortex, i.e., the HG, using the visual cortex, i.e., the forceps major (FM) as an internal control.

## Materials and Methods

### Participants

This was a study of children with CSNHL and healthy controls with normal hearing who underwent brain MRI scans with acquisition of DTI data. The eligibility criteria included the following: children between 3.5 and 18 years of age with profound CSNHL, a mean brain stem response threshold of >91 dB, auditory steady-state evoked potential >80 dB, hearing loss within the range of 250–4,000 Hz, and grossly normal MRI results for the brain and inner ear with no evidence of major structural abnormalities. None of the children with CSNHL had a history of using hearing aids upon enrollment. The exclusion criteria were the following: severe neurological disorders such as epilepsy, congenital leukodystrophy, or severe cognitive impairment such as autism or hyperkinetic syndrome, and poor-quality MRI scans that were rendered suboptimal for data analysis.

This study was carried out in accordance with all institutional, local, and national guidelines. Our study was approved by the institutional review board of our hospital. The parents or legal guardians of the children gave written informed consent in accordance with the Declaration of Helsinki. Statistical analysis was performed on the demographic information of the participants including age and gender. For continuous variable such as age, a two-sample *t*-test was used. For categorical variables such as gender, Fisher’s exact tests were used. A two-sided *p*-value less than 0.05 was considered as statistically significant.

### MRI Image Acquisition

The MRI images were acquired with a Siemens Magnetom Verio 3T MRI scanner (Siemens Healthcare; Erlangen, Germany) using a 12-channel phased-array head coil (Siemens). The head position was stabilized with sponge support. All study participants were sedated for the MRI scan with oral chloral hydrate under the care of medical professionals. There were no complications with regard to the oral sedatives.

A three-dimensional magnetization-prepared rapid gradient-echo (MP-RAGE) sequence was obtained with the following protocol: slice thickness, 0.9 mm; slice interval, 0 mm; repetition time, 1,700 ms; echo time, 2.9 ms; inversion time, 900 ms; matrix, 256 × 256; field of view, 220 × 220 mm^2^; and slice number, 140. The DTI scanning protocol was as follows: slice thickness, 2 mm; slice interval, 0 mm; slice number, 60; repetition time, 9,000 ms; echo time, 93 ms; and imaging matrix, 128 × 128. Motion-probing gradients were applied along 30 non-collinear directions with a *b* factor of 1,000 s/mm^2^ after an acquisition without diffusion weighting (*b* = 0 s/mm^2^). Additional parameters included the following: diffusion-sensitive gradient direction, 30; field of view, 220 × 220 mm^2^; number of excitations, 2.

### Diffusion Tensor Imaging Preprocessing and Tract-Based Spatial Statistics Analysis

**FIGURE 1:**
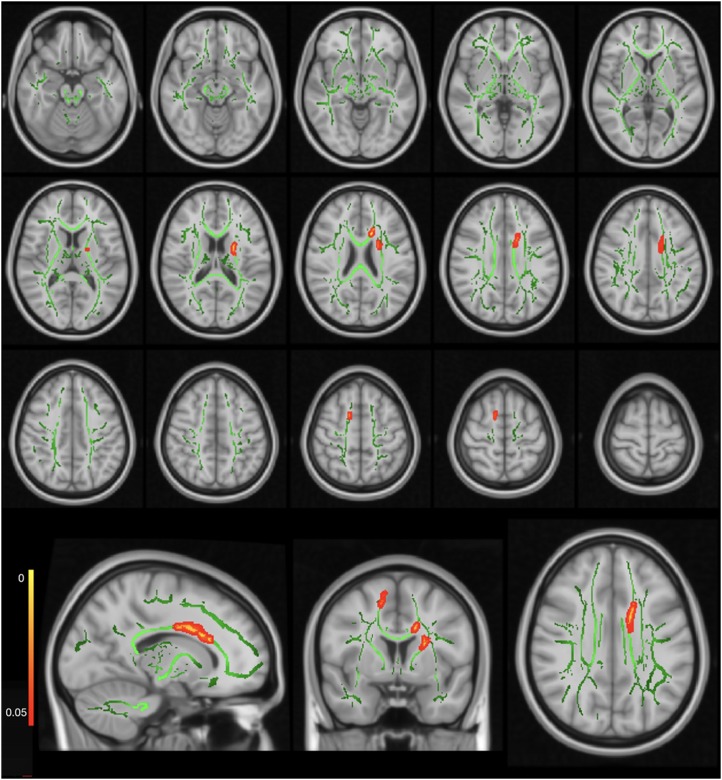
Axial diffusivity (AD) map showing significantly higher AD values in the CSHNL group as compared to the control group. The red–yellow highlighted areas indicate higher AD values in the right corticospinal tract, left anterior thalamic radiation, and left corpus callosum. The background image consists of the standard MNI T1-weighted template at 1-mm thickness and the FA skeleton (*green*). The images at the bottom row from the left side to the right side indicate sagittal, coronal, and axial images for one representative axial level.

**FIGURE 2:**
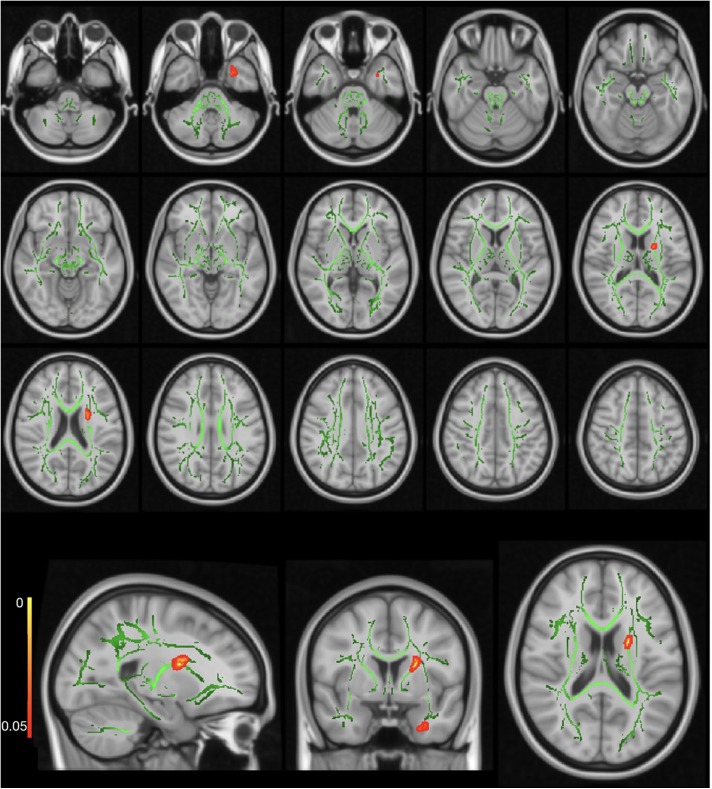
Radial diffusivity (RD) map showing significantly higher RD values in the CSHNL group as compared to the control group. The red–yellow highlighted areas indicate higher RD values in left inferior fronto-occipital fasciculus (IFOF) and left anterior thalamic radiation. The background image consists of the standard MNI T1-weighted template at 1-mm thickness and the fractional anisotropy (FA) skeleton (*green*). The images at the bottom row from the left side to the right side indicate sagittal, coronal, and axial images for one representative axial level.

The FMRIB Software Library (FSL) was used for DTI data preprocessing (version 5.0; FMRIB, Oxford, United Kingdom)^1^. TBSS^2^ was used to perform voxel-wise statistical analysis (Smith et al., 2006). First, the raw diffusion datasets were corrected for eddy current effects (Andersson and Sotiropoulos, 2016). Then, we used the Brain Extraction Tool for brain extraction (Smith, 2002). Subsequently, we fitted the preprocessed diffusion data into a tensor model to create the FA images (Basser et al., 1994). A non-linear registration was used to align the FA data from all the subjects into a common space, the Montreal Neurological Institute (MNI) MNI152 space (Fonov et al., 2009, 2011). A study-specific FA template was built as the target FA image for our study cohort, which was younger than 18 years. A mean FA image of all subjects was created according to a previously described method (Andersson et al., 2007). This mean FA image was then thinned to create a mean FA skeleton, which represented the centers of all the tracts common to all subjects. Each subject’s aligned data were then projected onto the MNI template image, and voxel-wise cross-subject statistics were applied to the data. The maps generated and analyzed were FA, mean diffusivity (MD), RD, and axial diffusivity (AD) according to a previously described method (Wheeler-Kingshott and Cercignani, 2009). λ1, λ2, and λ3 were the three eigenvalues of diffusion, while λ1 was also called AD (Figure 1), and averaging the maps of λ2 and λ3 represented RD (Figure 2).

Voxel-wise statistical analyses were performed using the FSL tool, Randomise^3^. This method implemented permutation-based inference (Wang et al., 2007). Age was treated as a covariant in the whole-brain TBSS analysis but not in the ROI analysis. Multiple comparison correction was performed using both the threshold-free cluster enhancement and cluster-based thresholding options (Smith and Nichols, 2009).

### Region of Interest Analysis Focusing on Auditory Cortex and Correlation Between Diffusion Tensor Imaging and Age

**FIGURE 3:**
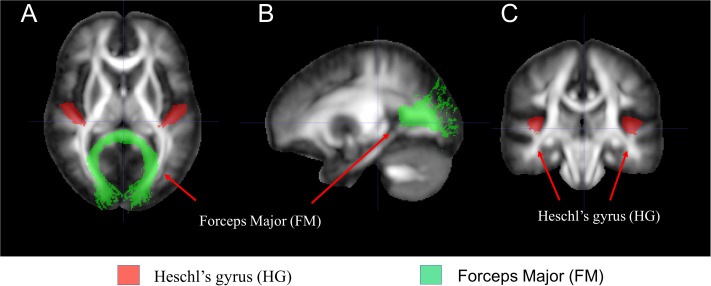
The white matter (WM) tracts of interest selected for region of interest (ROI) analysis. **(A)** Axial, **(B)** sagittal, and **(C)** coronal images of the selected brain regions.

Additional DTI analysis was performed with an ROI approach focusing on the auditory cortex, i.e., the WM tracts leading to HG, using the visual cortex, i.e., the FM of corpus callosum connecting the bilateral visual cortex as internal control as seen in Figure 3. We extracted the mean DTI values from HG and FM. Two-sample *t*-test was used to compare between-group differences in the mean FA, AD, and RD values for each tract of interest. The *p* < 0.05 was considered statistically significant. Correlations between mean FA, AD, and RD values and age in each group were obtained with the ROI methods focusing on HG while using FM as an internal control by computing pairwise Pearson correlation coefficients and the associated *p*-values before and after stratification of each group with a cutoff age of 7 years. The CSNHL group was then divided into two subgroups, i.e., the CSNHL group < 7 years and the CSNHL group ≥ 7 years, and the control group was divided into two subgroups in a similar manner with a cutoff age of 7 years. A value of *p* < 0.05 was considered statistically significant after Bonferroni correction for multiple comparisons.

## Results

### Demographic Information

A total of 34 children over 3.5 years old with profound CSNHL were enrolled in the study between February 1, 2017, and October 1, 2018, and each participant underwent brain MRI scanning for acquisition of DTI data and 3D T1-weighted sequences. Eleven children were subsequently excluded from the study: six children had major neurological diseases or major cognitive impairment, and five children with CSNHL had poor-quality MRI brain scans that prevented optimal imaging analysis. The final study cohort consisted of 23 children with profound CSNHL (11 boys and 12 girls; mean age ± SD: 7.21 ± 2.67 years; range: 4.1–13.5 years). The control group included 18 age-matched children (11 boys and 7 girls; mean age ± SD: 10.86 ± 3.56 years; range: 4.5–15.3 years). All study participants in both the CSNHL group and the control group were right-handed (Table 1).

**Table 1:** Summary of characteristics of study participants.

	CSNHL Group (*n* = 23)	Control Group (*n* = 18)
Age DTI obtained (years)	7.21 ± 2.67 (4.1–13.5)	10.86 ± 3.56 (4.5–15.3)
Age CSNHL diagnosed (years)	0.77 ± 0.18 (0.5–1.17)	∖
Gender	11 Male/12 Female	11 Male/7 Female
Handedness	all righthanded	All righthanded
dB hearing loss: Left ear	108.45 ± 7.59 (94–120dB)	∖
dB hearing loss: Right ear	106.62 ± 8.06 (91–120dB)	∖

*Age (years) are presented as mean age ± SD; hearing loss in left ear and right ear are presented as mean dB ± SD. dB: decibels.*

No significant differences between the CSNHL group and the control group were noted for gender (*p* = 0.60). Regarding age, the CSNHL group was significantly younger than the control group (*p* = 0.0006).

### Whole-Brain Voxel-Wise Tract-Based Spatial Statistics Data

The whole-brain TBSS analysis showed no significant differences between the CSNHL and control groups for either the FA or the MD map (*p*-corrected > 0.05). Significantly higher AD values were observed in the left anterior thalamic radiation, right corticospinal tract, and left corpus callosum in the CSNHL group, as compared to the control group (*p*-corrected < 0.05) (Table 2). The coordinates of the local maxima and cluster sizes are presented in Figure 1 and Table 2. There were significantly higher RD values for the left anterior thalamic radiation and left IFOF (*p*-corrected < 0.05 for both threshold-free cluster correction and cluster-based thresholding correction) in the CSNHL group, relative to the control group, as shown in Figure 2 and Table 2.

**Table 2:** Summary of DTI parameters showing significant differences between the CSHNL group and the Control Group.

	Voxels	*P* value	MNI Coordinates			Atlases (Tract from JHU)
			*X*	*Y*	*Z*	
AD	225	<0.001	102	144	93	Corticospinal tract R
	133	0.007	108	123	84	Anterior thalamic radiation L
	105	0.022	75	131	122	Corpus callosum L
RD	103	0.016	115	134	31	IFOF L
	81	0.037	109	126	86	Anterior thalamic radiation L

*JHU, Johns Hopkins University; L, Left; R, Right.*

### Region of Interest Data Focusing on Auditory Cortex and Correlation Between Diffusion Tensor Imaging and Age

**FIGURE 4:**
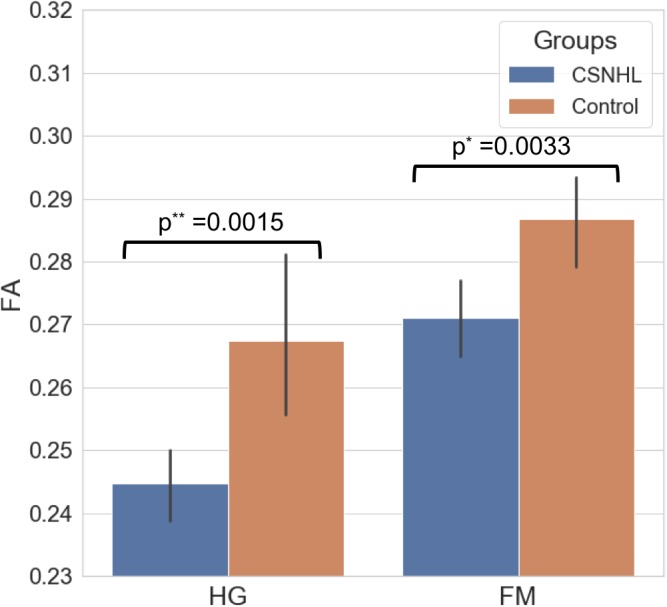
The mean FA values for the HG and the FM in both the CSNHL group and the control group.

There were statistically significant lower FA values in the CSNHL group compared to that in the control group in the HG (*p* = 0.0015) and FM (*p* = 0.0033) as presented in Table 3 and Figure 4.

**Table 3:** DTI parameters for the region of interest (ROI) analysis focusing on the Heschl’s gyrus (HG) while using the forceps major (FM) as an internal control.

		CSNHL Group	Control Group	*p*
		*n* = 23	*n* = 18	
		Mean (SD)	Mean (SD)	
FA	HG^∗^	0.24 (0.01)	0.27 (0.03)	<0.01
	FM^∗∗^	0.27 (0.02)	0.29 (0.02)	<0.01
RD	HG	0.87 (0.02)	0.85 (0.03)	0.08
	FM	0.98 (0.03)	0.99 (0.04)	0.66
AD	HG	0.98 (0.03)	0.97 (0.03)	0.15
	FM	1.13 (0.04)	1.15 (0.04)	0.32

*^∗^p = 0.0015: when comparing the mean FA value of HG between the CSNHL group and the Control Group. ^∗∗^p = 0.0033: when comparing the mean FA value in the FM between the CSNHL group and the Control Group. Values indicate the mean values in each parameter. FA: fractional anisotropy, RD: radial diffusivity, AD: axial diffusivity.*

**FIGURE 5:**
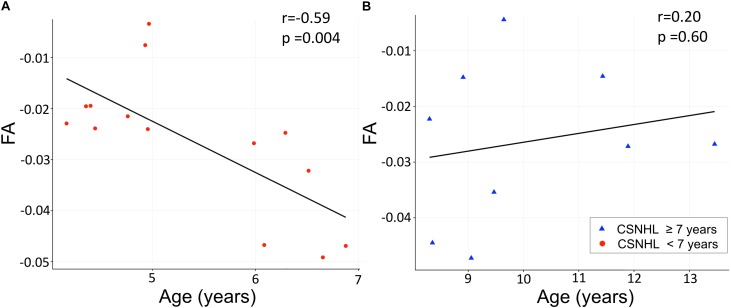
Correlation analysis between the mean FA values and age. The mean FA values on the *Y*-axis indicate the differences between the Heschl’s gyrus (HG) and the forceps major (FM). **(A)** Correlation between the mean FA values and age in the CSNHL group < 7 years of age. **(B)** Correlation between the mean FA values and age in the CSNHL group ≥ 7 years of age.

There was statistically significant negative correlation between the mean FA values in the HG relative to the FM (as an internal control) and age in the CSNHL group < 7 years (*n* = 14, *r* = −0.59, *p* = 0.004), while no similar correlation was noted in the CSNHL group ≥ 7 years (*n* = 9, *r* = 0.20, *p* = 0.60). While in the control group stratified by the age of 7 years, there were no statistically significant correlation with age either in the control group < 7 years (*n* = 3, *r* = 0.99, *p* = 0.10) or in the control group ≥ 7 years (*n* = 15, *r* = 0.45, *p* = 0.09) as presented in Figure 5.

## Discussion

In this study, we found higher AD and RD values in the children with CSNHL, compared to the healthy children with normal hearing. These differences were present in several WM tracts, including the left anterior thalamic radiation, right corticospinal tract, corpus callosum, and left IFOF. The higher AD values indicated the presence of WM microstructural alterations in axonal formation, and the higher RD values indicated altered myelination in the children with CSNHL. To the best of our knowledge, our study was the first to report differences in the AD and RD values in children with CSNHL between 4.1 and 13.5 years of age. Moreover, our ROI analysis focusing on the auditory cortex showed lower FA values in the HG and negative correlation between the FA values and age up to 7 years in the children born deaf.

Tract-based spatial statistics is a commonly used program for comparing groups on a voxel-by-voxel basis to identify differences in FA and other DTI parameters (Maller et al., 2014). Using TBSS, we performed a whole-brain analysis of normalized brain to evaluate the WM microstructure. Our TBSS approach allowed for a broad characterization of between-group differences with stringent statistical corrections (Smith and Nichols, 2009). FA and MD are the two common diffusion indicators for DTI data, and they have been used to evaluate WM properties in various diseases, such as prelingual deafness and traumatic brain injury (Le Bihan et al., 2001; Hribar et al., 2014; Park et al., 2018). FA values are related to the WM microstructural integrity and directionality of axonal fibers within a voxel (Assaf and Pasternak, 2008). MD is the mathematical combination of RD and AD. Nevertheless, assessing the diffusion properties such as AD and RD in regions parallel to and perpendicular to WM tracts may provide additional information about brain microstructure such as axonal formation and the presence of myelin (Song et al., 2002, 2005; Counsell et al., 2006; Sun et al., 2006), reflecting distinct aspects of WM microstructure. Directional diffusivity shows great potential to be specific biomarkers for injury to the axons and myelin (Xu et al., 2008), and DTI makes it possible to evaluate microstructural diffusivity (Hribar et al., 2014). In our study, we evaluated WM microstructural properties by analyzing RD and AD along with FA and MD. Although we found no significant differences in FA or MD values between the CSNHL group and the control group in the whole-brain TBSS analysis, our finding of higher RD and AD values in specific WM regions indicated the presence of WM microstructural differences in the children with CSNHL compared to the children with normal hearing.

The published literature is conflicting regarding the WM microstructural properties in the congenital deaf people. Various inconsistent structural differences in both auditory and non-auditory areas have been reported in the literature. This might be due to different analytical approaches with some studies using a whole-brain voxel-wise TBSS approach while others focusing on the specific regions of interests such as the auditory cortex. In addition, some studies adopted a combined approach using both the whole-brain TBSS and ROI methods, and some used a volumetric method to analyze the volumes of the auditory cortex (Emmorey et al., 2003; Park et al., 2018). For instance, a human study using a volumetric approach showed less WM in the HG leading to larger gray matter–WM ratio in the congenitally deaf adults compared to the hearing adults (Emmorey et al., 2003). Their findings suggest that lack of auditory stimuli from birth may lead to less myelination and possibly fewer fibers connected to the auditory cortices. A study with cortical cytoarchitectural analysis showed morphological changes in the auditory cortex with thinner auditory fields in a deaf animal research (Berger et al., 2017).

Non-auditory effects of congenital deafness have also been reported including the visual cortex, somatosensory projections, and motor tracts (Hribar et al., 2014; Kral et al., 2019). We speculate the non-auditory changes found in our whole-brain TBSS voxel-wise analysis might be due to the following reasons. First, neuroplasticity may occur in the children with CSNHL. The developing brain may exert compensatory cross-modal plasticity with reorganization of other sensory modalities such as visual ability, thus demonstrating a phenomena described in the literature as a visual “take over” of auditory areas in deaf children (Kral et al., 2019). In addition, sensory and motor inputs are linked to each other during development, and motor cortices may mediate the increased auditory-evoked activity to sounds in humans (Reznik et al., 2015). Therefore, it is not surprising to find motor tracts such as the corticospinal tracts showing higher AD and RD values in our cohort with CSNHL compared to the hearing children. Second, our relatively small sample size consisted of the children ranging from 4.1 to 13.5 years of age, which was different from some studies in the literature (Miao et al., 2013; Park et al., 2018). In addition, our study was limited by the fact that our two study groups were not matched by age with the CSNHL group being younger than the control group. All these factors may decrease the sensitivity of detecting subtle differences in the DTI measures. Third, heterogenous FA images are commonly seen in children’s developing brains. Therefore, there is an increased risk for false-positive results, which have been noted in the published literature (Smith et al., 2006). Nevertheless, our additional analysis with the ROI approach focusing on the auditory cortex showed the HG was affected in the CSNHL group with lower FA values compared to the hearing children. As for our future study, we will perform volumetric analysis of the auditory cortex and resting-state functional connectivity to further understand the brain structural and functional differences between the children with CSHNL and the children with normal hearing.

Some of our study results were consistent with the published literature, while some were divergent from prior reports. We found no significant differences for FA or MD values between the CSNHL group and the control group in the whole-brain TBSS analysis. One prior study showed reduced FA values but unchanged MD values along the auditory pathways of patients with sensorineural hearing loss when compared to the controls. Another study, which used TBSS to study prelingually deaf children, found no significant differences between the FA maps of patients and controls, but positive correlations between FA values and age for the deaf children in almost all WM tracts. Differences in age may partially explain the differences between our study results and those of the studies by Chang et al. (2004) and Park et al. (2018). The study by Park et al. (2018) included patients from 1 to 7 years old, while our study participants with CSNHL were between 4.1 and 13.5 years old. In their additional analysis focusing on children over 4 years old, they found no significant differences between the deaf and control children for the DTI parameters, which was generally in line with our finding. Our study enrolled the children with CSNHL over 3.5 years old for two reasons. First, prior studies have shown that the brain changes the fastest from 1 to 3 years of age (Hermoye et al., 2006; Tierney and Nelson, 2009). Studying the developing brain during this period is challenging because the brain is small but rapidly growing. The regional topology and myelination also change during this period. Furthermore, brain function might not match the anatomy precisely at this stage of brain development (Fan et al., 2011). In addition, although 3.5 years of age is an optimal time for language function and brain development (Sharma et al., 2002, 2015), it is challenging to enroll study participants at this age. Some families did not seek medical treatment such as cochlear implant until the children were older enough for kindergarten or elementary school. In our experience, this delay in treatment was more commonly seen in rural areas with limited social and financial support. We therefore designed our study targeting the children with CSNHL between 3.5 and 18 years because this age group was particularly vulnerable and was at a disadvantage for learning in school due to hearing impairment. Because of the children’s ages in our study cohort, it was expected to see our study results different from others targeting different ages of children with hearing loss.

Our additional analysis of age relationships in the WM microstructure showed significant correlation between the mean FA values and the age in the deaf children younger than 7 years. These findings implicate the WM microstructural alterations occur in younger children but may be compensated in older children with CSNHL. WM maturation occurs mostly in the first 12 months of life and plateaus after 24 months of age (Muftuler et al., 2012). Our study participants were all older than 2 years, and therefore, most of the major developmental changes should have already occurred in our cohort. Nevertheless, we assumed that some minor WM alterations may still be ongoing in our cohort. This assumption was supported by prior reports indicating gradual increases of FA values in various WM tracts as the brain matures from childhood to adulthood (Muftuler et al., 2012). However, we did not detect the expected age effect in our study with the whole-brain TBSS analysis. We believe this study result might be due to the wide age range in our study cohort, the two groups not age matched, and the relatively small sample size. Nevertheless, our additional ROI analysis focusing on the auditory cortex showed an age effect in the CSNHL group with significant negative correlation between the mean FA values in the HG and age in the subgroup of deaf children younger than 7 years.

We found that the WM microstructural differences were located predominantly in the frontal brain, including the anterior thalamic radiation connecting the anterior thalamus with prefrontal areas, and IFOF connecting the occipital lobe to the temporal lobe and frontal lobe. Frontal brain is associated with cognitive function such as executive function, speech, and language development. There are positive correlations between cognitive function and refined fiber organization of WM tracts (Muftuler et al., 2012). In addition, studies have shown an association between trajectories of brain WM maturation and intellectual performance (Tamnes et al., 2010). Therefore, our study findings of WM differences in the frontal brain implicate potential association between WM microstructural properties and cognitive function in children with CSNHL. We hypothesize that the detrimental effects on the WM microstructures as reflected by lower FA in young deaf children may represent potential neural correlates of cognitive development in the children with CSNHL and may contribute to their developmental delay. We recognize that this hypothesis was based on our speculation since we did not collect data to assess developmental delay or cognitive development for the current study. Nevertheless, our study has clinical relevance indicating preferential frontal WM involvement in the vulnerable children with hearing loss, which may serve as preliminary data for hypothesis generating for future large-scale studies of CSNHL, cognitive functioning, and developmental delay.

Our study showed higher values for AD and RD in the left anterior thalamic radiation in the children with CSNHL compared to the hearing children. The acoustic radiation is a component of the anterior thalamic radiation, and it is critical for hearing. This fiber tract originates in the medial geniculate nucleus and terminates in the primary auditory cortex, i.e., the HG (Wakana et al., 2004). Our study finding of significantly higher values in both AD and RD indicates loss of WM microstructural integrity of the acoustic radiation, as reported in another study (Husain et al., 2011). The higher value in AD implicates axonal abnormalities, intrinsic neuronal degradation, and morphologic differences in neuronal fibers (Lin et al., 2008; Profant et al., 2014). The higher value in RD may reflect abnormal myelination such as demyelination or dysmyelination (Song et al., 2002, 2005; Hasan et al., 2008), or it could reflect a higher number of crossing fibers, as shown in a study of congenital deafness in adults (Karns et al., 2017). For the children with CSNHL in our study, we speculate that the higher AD values may be due to abnormal axonal maturity such as less axonal density and caliber, while the higher RD might be due to abnormal myelination such as loss of myelin integrity.

Our study showed higher AD and RD in the corticospinal tract, corpus callosum, and IFOF in children with CSHNL as compared to the hearing children. These brain structures regulate motor, language, and visual function (Vanderah, 2016; Wu et al., 2016). The corticospinal tract is a collection of axons that carry motor-related information from the cerebral cortex to the spinal cord (Vanderah, 2016). Prior studies have found that hearing-impaired children with sensory organizational deficits have poor balance and have presented with motor problems (Crowe and Horak, 1988; Hartman et al., 2011). Our study findings of higher AD in the corpus callosum of the children with CSNHL is in agreement with prior studies of deaf individuals (Kim et al., 2009). The corpus callosum is responsible for transmitting neural messages between the right and left hemispheres and plays an important role in transmitting auditory, visual, and linguistic signals. Therefore, it is not surprising to see different diffusivity values in the corpus callosum in the children with CSNHL compared to the hearing children in our cohort, who may undergo brain reorganization from the lack of sound stimuli.

There are several limitations to our study. First, our study cohort of patients with CSNHL had a wide age range, from 4.1 to 13.5 years of age, and were not age matched with the control group, which may confound the findings in our study. Moreover, we did not evaluate the CSNHL patients younger than 4 years, which may limit the generalizability of our study results to all children with CSHNL. Second, our sample size was not large enough to stratify the patients based on their clinical and lifestyle factors, such as knowledge of sign language and the extent of special education for the hearing impaired, which may alter brain structure and function. Third, our TBSS method involved whole-brain analysis with stringent statistical comparisons, which may limit the power to detect subtle differences in specific brain regions between the two groups. Other methods, such as the ROI approach, may help us to evaluate differences in the DTI parameters in the particular regions of WM that are relevant to the auditory pathway (Karns et al., 2017). Therefore, we performed additional ROI analysis focusing on the auditory cortex. Fourth, although DTI is commonly used to study the WM microstructure, it has its own inherent limitations as an imaging method. For instance, the tensor model only represents one major fiber direction in a voxel, so DTI analysis could potentially be confused by cross-fiber regions (Behrens et al., 2007). Last, our study was limited due to the lack of analysis on the sedation effect. We used chloral hydrate to induce a light-sedated state because it was easy to use as a liquid for oral intake for children, and it had a low adverse respiratory effect. However, all children in our study were sedated during the MRI scans. We therefore could not perform an analysis of sedation effect by comparing the group with sedation with the group without sedation. Nevertheless, we recognize that sedation may affect brain structure and function. A brain connectivity analysis of resting-state functional MRI study examined the effect of chloral hydrate-induced light sedation in school-aged children (Wei et al., 2013). Their study showed a global detrimental effect of sedation on the functional interactions of the brain, especially on the information-processing properties. We will incorporate the sedation effect in our future studies of brain structure and function in children with CSNHL.

In summary, we assessed the cerebral WM microstructural properties in children with CSNHL. We found higher AD and RD values in the left anterior thalamic radiation, right corticospinal tract, corpus callosum, and left IFOF in the children with CSNHL compared to the hearing children. We also found lower FA values in the auditory cortex in the deaf children compared to the hearing children. Furthermore, we identified the FA values being negatively correlated with age up to 7 years in the children born deaf. We speculate that the WM microstructural alterations in the children with CSNHL could be due to injury of the auditory pathway or brain functional neuroplasticity with reorganization. This information may help to design and track hearing rehabilitation in the children with CSNHL.

## Data Availability

The raw data supporting the conclusion of this manuscript will be made available by the authors, without undue reservation, to any qualified researcher.

## Ethics Statement

This study was carried out in accordance with all institutional, local, and national guidelines. Our study was approved by the institutional review board of the First Affiliated Hospital of Guangxi Medical University. The parents or legal guardians of the children gave written informed consent in accordance with the Declaration of Helsinki.

## Author Contributions

MJ and ZW contributed equally to this work. MJ and LL designed the study. MJ, BC, CW, NY, and CZ contributed to manuscript preparation and revision. ZW performed data acquisition and analysis. MJ, ZW, CW, NY, and BC performed data interpretation. LL, BC, and CZ were responsible for the study supervision. All authors approved the final manuscript.

## Conflict of Interest Statement

The authors declare that the research was conducted in the absence of any commercial or financial relationships that could be construed as a potential conflict of interest.

## Footnotes

1 http://www.fmrib.ox.ac.uk/fsl

2 https://fsl.fmrib.ox.ac.uk/fsl/fslwiki/TBSS/

3 https://fsl.fmrib.ox.ac.uk/fsl/fslwiki/Randomise
